# 

**Published:** 1882-01

**Authors:** 


### CONTENTS.

***MISCELLANEOUS.***

PAGE.

Alternation of Remedies.—Editorial. . . . . . . . . 9
Unification: A Fatal Error. Ad. Lippe, M. D., . . . . . . 14
Homœopathic Therapeutics and Pathological Anatomy. P. P. Wells, M. D., . . . . . . . . . . . . . . 18
Clinical Bureau of Am. Inst. of Homœopathy. T. F. Pomeroy, M. D., 32
Notes from our Scrap-Book. E. J. L., . . . . . . . . 33
Criticisms on the Treatment of the late President. [Extracts.] . . 36
Hygienic Aphorisms, . . . . . . . . . . . 41

***CLINICAL BUREAU:***

Progressive Pernicious Anæmia: A Case with Autopsy. Edward Cranch, M. D., . . . . . . . . . . . . 43

Clinical Reflections. Ad. Lippe, M. D., . . . . . . 45

Clinical Cases. E. W. Berridge, M. D., . . . . . . . 47

Similia not Idem. Hahnemann's Opinion. . . . . . . 50

Periscope, . . . . . . . . . . . . . 50

***BOOK REVIEWS, NOTICES, ETC.***

Special Pathology and Diagnostics. By C. G. Raue, M. D., . . . 54
Insanity and its Treatment. By Samuel Worcester, M. D., . . . 55
Proceedings of the Hom. Med. Society of Ohio, . . . . . . 56
Journal of European Travels. C. Pearson, M. D., . . . . . 56
New Books, . . . . . . . . . . . . . 56

